# Long-term effects of abomasal infusion of linoleic and linolenic acids on the enrichment of n-6 and n-3 fatty acids into milk fat of lactating cows

**DOI:** 10.3168/jdsc.2024-0627

**Published:** 2024-12-16

**Authors:** J.M. dos Santos Neto, L.C. Worden, J.P. Boerman, C.M. Bradley, A.L. Lock

**Affiliations:** Department of Animal Science, Michigan State University, East Lansing, MI 48824

## Abstract

Our objective was to compare abomasal infusions of linoleic (18:2n-6) and α-linolenic (18:3n-3) acid on the incorporation of n-6 and n-3 fatty acids (FA) into the milk fat of dairy cows and to evaluate their potential carryover effects. Six rumen-cannulated multiparous Holstein cows (252 ± 33 DIM) were fed the same diet and assigned to 1 of 2 treatments in a completely randomized design with repeated measures. Treatments were abomasal infusions (67 g/d total FA) of (1) n-6 FA blend (N6) to provide ∼43 g/d 18:2n-6 and 8 g/d of 18:3n-3; or (2) n-3 FA blend (N3) providing 43 g/d 18:3n-3 and 8 g/d 18:2n-6. The treatment period lasted from d 1 to 20, and the carryover period lasted from d 21 to 36. Compared with N6, the N3 treatment increased the yields of total n-3 FA, 18:3n-3, and eicosapentaenoic acid (20:5n-3) from d 4 to 20, decreased total n-6 FA from d 8 to 20 and 18:2n-6 from d 8 to 16, and tended to decrease arachidonic acid (20:4n-6) from d 12 to 16. During the treatment period, n-3 FA transfer efficiency to milk for N3 was 47%, whereas n-6 FA transfer efficiency to milk for N6 was 39%. A similar pattern was observed for milk FA content, with N3 increasing the contents of total n-3 FA, 18:3n-3, and 20:5n-3 from d 4 to 20; and decreasing total n-6 FA from d 4 to 20, 18:2n-6 from d 4 to 16, and 20:4n-6 from d 12 to 16 compared with N6. We only observed positive carryover effects for N3, with the treatment increasing or tending to increase the yield of n-3 FA until d 28 and increasing the content of total n-3 FA until d 26. We observed no carryover effects for N6. In addition, we did not detect 22:6n-3 either during the treatment or carryover periods. In conclusion, abomasally infusing N3 and N6 for 20 d increased the yields and contents of n-3 and n-6 FA in milk fat, respectively. Interestingly, the increases were more pronounced in n-3 than in n-6 milk FA, with a transfer efficiency of 47% and 39% during the treatment period, respectively. Furthermore, a positive carryover effect was observed only for n-3 FA.

## Highlights

• Abomasally infusing 18:3n-3 increased n-3 FA in milk fat.
• Positive carryover effects were observed for n-3 milk FA.
• We did not detect 22:6n-3 in milk.

## Graphical Abstract

**Figure:**
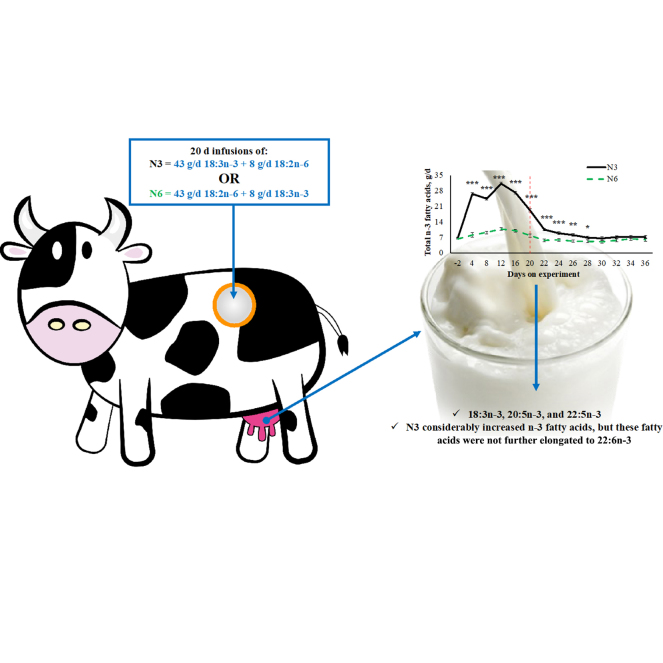
**Summary:** Mammals do not synthesize essential fatty acids (FA) vital for proper physiological functioning; thus, these FA must be provided in the diet. The essential FA linoleic (18:2n-6) and a-linolenic acids (18:3n-3) are partially converted to longer-chain FA: 18:2n-6 to arachidonic acid (20:4n-6), and 18:3n-3 to eicosapentaenoic acid (20:5n-3) and docosahexaenoic acid (22:6n-3). We compared abomasal infusions of 18:2n-6 (N6) and 18:3n-3 (N3) on the incorporation of essential FA into the milk fat of cows. Infusions lasted 20 days, with subsequent carryover evaluation until day 36. During the treatment period, n-3 FA transfer efficiency to milk for N3 was 47%, whereas n-6 FA transfer efficiency to milk for N6 was 39%. In addition, only n-3 FA had a positive carryover effect. However, the absolute quantity of n-3 FA in milk fat was still very low, especially considering 20:5n-3 and the lack of 22:6n-3.

Linoleic (18:2n-6) and α-linolenic (18:3n-3) fatty acids (**FA**) are essential for dairy cows because mammals lack the enzymes Δ^12^- and Δ^15^-desaturase capable of inserting double bonds distal to position 10 of the carbon chain (Palmquist, 2009). These PUFA can be further elongated to longer-chain PUFA, with 18:2n-6 being converted to arachidonic acid (20:4n-6) and 18:3n-3 to eicosapentaenoic acid (20:5n-3) and docosahexaenoic acid (22:6n-3; Palmquist, 2009). Both n-6 and n-3 FA are vital for several biological processes, such as being constituents of cell membranes, synthesizing eicosanoids, and modulating the immune system (Palmquist, 2009; Moallem, 2018). However, although 20:4n-6 can be converted to proinflammatory eicosanoids, n-3 FA and their products have the potential to reduce excessive inflammatory responses (Palmquist, 2009). Thus, some authors have proposed that dietary supplementation of n-3 FA to dairy cows could also benefit consumers, as milk and its derivatives are important and popular components of Western diets (Moallem, 2018).

Ruminal biohydrogenation is the primary factor challenging the enrichment of PUFA in milk. This process has several steps, with the general outcome being the extensive conversion of unsaturated to saturated FA by rumen bacteria (Jenkins et al., 2008). In addition to biohydrogenation, the storage and transport of absorbed n-6 and n-3 FA play a crucial role in determining the transfer of PUFA to milk. Lipid fractions such as phospholipids (**PL**) and cholesterol-ester (**CE**) less efficiently supply FA to the mammary gland compared with triglycerides (**TG**) and nonesterified fatty acids (**NEFA**; Christie, 1981).

Abomasally infusing PUFA avoids rumen biohydrogenation, allowing for more detailed examination of postabsorptive metabolism of these FA. In our companion paper, we reported the effects of abomasally infusing 18:2n-6 and 18:3n-3 on the incorporation of n-6 and n-3 FA into plasma lipid fractions of lactating dairy cows and evaluated their potential carryover effects (dos Santos Neto et al., 2024). In this paper, our objective was to compare the effects of these same infusions on the incorporation of n-6 and n-3 FA into the milk fat of dairy cows and evaluate their potential carryover effects.

The animals and treatments in this study are the same as those reported in our companion paper (dos Santos Neto et al., 2024). Experimental procedures were approved by the Institutional Animal Care and Use Committee at Michigan State University (East Lansing, MI). Six rumen-cannulated multiparous Holstein cows averaging 252 ± 33 DIM and 44 ± 6 kg milk/d (mean ± SD) from the Michigan State University Dairy Cattle Teaching and Research Farm (East Lansing, MI) were randomly assigned to one of 2 treatments in a completely randomized design with repeated measures. Before the start of the experiment (−2 d), pre-treatment measurements were taken, and abomasal infusion devices were inserted into the abomasum as described previously (Tyburczy et al., 2008). Treatments were abomasal infusions (67 g/d total FA) of (1) n-6 FA blend to provide approximately 43 g/d 18:2n-6 and 8 g/d of 18:3n-3 (**N6**); or (2) n-3 FA blend to provide 43 g/d 18:3n-3 and 8 g/d 18:2n-6 (**N3**). The choice of doses, the proportions of safflower, high linolenic flaxseed, and palm oil used, and the FA profile of treatment blends are described in detail in dos Santos Neto et al. (2024). The capitalized acronyms N6 and N3 are used only when referring to the abomasal infusion of n-6 and n-3 FA blends in the current study. The N6 and N3 treatment blends were prepared using safflower, high 18:3n-3 flaxseed, and palm oil. The oils were saponified to ensure infusions were supplied as FA rather than triglycerides (dos Santos Neto et al., 2024). Daily doses of N6 and N3 were weighed in individual glass jars and dissolved in ethanol, resulting in an infusate solution of 108 mL. The infusate solution was divided into 4 equal infusions per day at 6-h intervals. Infusate solutions were delivered into infusion lines using 60-mL plastic syringes. This treatment period lasted from d 1 to 20. After d 20, all cows remained on the same diet but did not receive abomasal infusions. This carryover period went from d 21 to 36. Cows were housed in individual tiestalls and milked twice daily (04:30 and 15:30). Throughout the treatment and carryover periods, all animals received a common diet (dos Santos Neto et al., 2024) formulated to meet their nutrient requirements (NRC, 2001).

The collection and analysis of diet ingredients and orts, as well as diet and N6 and N3 formulations, are described in our companion paper (dos Santos Neto et al., 2024). Access to feed was locked from 08:00 to 10:00 for collection of orts and offering of the new feed. Feed intake was recorded, and cows were offered 115% of the expected intake at 10:00 daily. Water was available ad libitum in each stall, and stalls were bedded with sawdust and cleaned twice daily. Pre-treatment DMI and yields of milk and milk components were determined on d −2 before the first infusion. During the treatment period, DMI and milk yield were determined daily, and milk samples were collected on d 4, 8, 12, 16, and 20 of infusions. During the carryover period, these variables were evaluated on d 22, 24, 26, 28. 30, 32, 34, and 36. Milk yield was recorded, and 2 milk samples were collected at each milking. Milk component and FA analysis were performed according to Bales and Lock (2024) and yields of individual FA (g/d) in milk fat calculated according to Benoit et al. (2024).

We performed separate statistical analyses for the treatment (from 4 to 20 d) and carryover periods (from 21 to 36 d); however, the final model for the treatment period was the same as that used for the carryover period (dos Santos Neto et al., 2024). Data were analyzed using the GLIMMIX procedure of SAS (version 9.4, SAS Institute Inc., Cary, NC) with repeated measures as Y_ijk_ = μ + F_i_ + T_j_ + C_k_ (F_i_) + F_i_ × T_j_ + pFM + e_ijk_, where Y_ijk_ = the dependent variable, μ = the overall mean, F_i_ = the fixed effect of treatment, T_j_ = the fixed effect of time, C_k_ (F_i_) = the random effect of cow within treatment, F_i_ × T_j_ = the fixed effect of the interaction between treatment and time, pFM = pre-treatment measurement used as a covariate, and e_ijk_ = the residual error. First-order autoregressive was the covariance structure used for analysis. To estimate milk FA yield response (**FAYR**) to additional FA intake, we used the predicted individual milk FA yield values adjusted for covariates. Milk FAYR was calculated for each sampling day during the treatment period for both n-6 and n-3 FA. For each day, FAYR-n-6 (%) = [(n-6 milk FA yield of individual cows on N6 − average n-6 milk FA yield of N3)/(n-6 FA infused in individual cows on N6 − average of infused n-6 FA of N3)], and FAYR-n-3 (%) = [(n-3 milk FA yield of individual cows on N3 − average of n-3 milk FA yield of N6)/(n-3 FA infused in individual cows on N3 − average of infused n-3 FA of N6)]. The model used to evaluate FAYR included time as the only fixed effect, and their respective intercepts were tested to determine if they were different from zero. Significance was declared at *P* ≤ 0.05 for main effects and *P* ≤ 0.10 for interactions. Tendencies were declared at *P* ≤ 0.10 for main effects and *P* ≤ 0.15 for interactions. We initially used the Bonferroni adjustment but removed it from the final analysis because it did not change the interpretation of the results.

During the treatment period, we did not observe any main effect of treatment on DMI or milk production (*P* ≥ 0.18), with no interactions between treatment and time, except for a tendency for an interaction for milk protein yield (*P* ≥ 0.13, data not shown). However, there were no differences between N3 and N6 within days (*P* ≥ 0.25). Overall, we observed the following means (± SD): 28.0 ± 4.44, 39.6 ± 3.78, 1.38 ± 0.27, and 1.26 ± 0.22 kg/d for DMI and the yields of milk, milk fat, and milk protein, respectively. We observed (mean ± SD) 3.49 ± 0.22 and 3.19 ± 0.33 g/100 g for the contents of milk fat and milk protein, respectively.

We did not observe interactions for milk FA sources on either a yield or content basis (*P* ≤ 0.17, Table 1). We observed interactions between treatment and time for milk FA yields of total n-3 FA, total n-6 FA, 18:2n-6, 18:3n-3, 20:4n-6, 20:5n-3 (*P* ≤ 0.06, Figure 1), and docosapentaenoic acid (22:5n-3; *P* = 0.04, data not shown). Compared with N6, N3 increased or tended to increase the yields of total n-3 FA (16.5 g/d), 18:3n-3 (15.8 g/d), 20:5n-3 (0.44 g/d, *P* < 0.01, Figure 1), and 22:5n-3 from d 4 to 20 (0.23 g/d, *P* ≤ 0.06, data not shown). Compared with N3, N6 increased or tended to increase total n-6 FA from d 8 to 20 (16.4 g/d), 18:2n-6 from d 8 to 16 (18.7 g/d), and 20:4n-6 from d 12 to 16 (*P* ≤ 0.08, Figure 1). We observed positive FAYR for n-3 FA during the treatment period (*P* < 0.01), with an overall transfer efficiency average of 47%. We also observed positive FAYR for n-6 FA during the treatment period (*P* < 0.01), with an overall transfer efficiency average of 39% (Figure 1).

**Table 1:** Milk fatty acid yields and contents of cows during treatment (from d 1 to 20) and carryover periods (from d 21 to 36)

Variable	Treatment^1^		SEM	*P*-value^2^		
	N6	N3		Trt	Day	Trt × Day
Yield, g/d						
Treatment period						
De novo	355	387	22	0.38	<0.01	0.22
Mixed	475	420	20	0.21	0.16	0.21
Preformed	440	508	44	0.33	<0.01	0.17
Carryover period						
De novo	372	382	72.5	0.94	0.78	0.60
Mixed	420	422	112	0.99	0.96	0.44
Preformed	476	507	78.9	0.81	0.67	0.55
Content, g/100 g						
Treatment period						
De novo	28.5	28.3	0.81	0.90	0.95	0.86
Mixed	33.5	35.2	0.39	0.08	0.35	0.61
Preformed	38.5	36.7	0.82	0.23	0.27	0.23
Carryover period						
De novo	28.3	28.9	0.25	0.40	0.88	0.84
Mixed	33.1	35.1	0.85	0.35	0.45	0.71
Preformed	37.8	36.6	0.86	0.43	0.16	0.35

1 Treatments were abomasal infusions of approximately 43 g/d of 18:2n-6 (N6) or 18:3n-3 (N3).

2 Trt = main effect of treatment; Day = main effect of day; Trt × Day = interaction between treatments and day.

**Figure 1:**
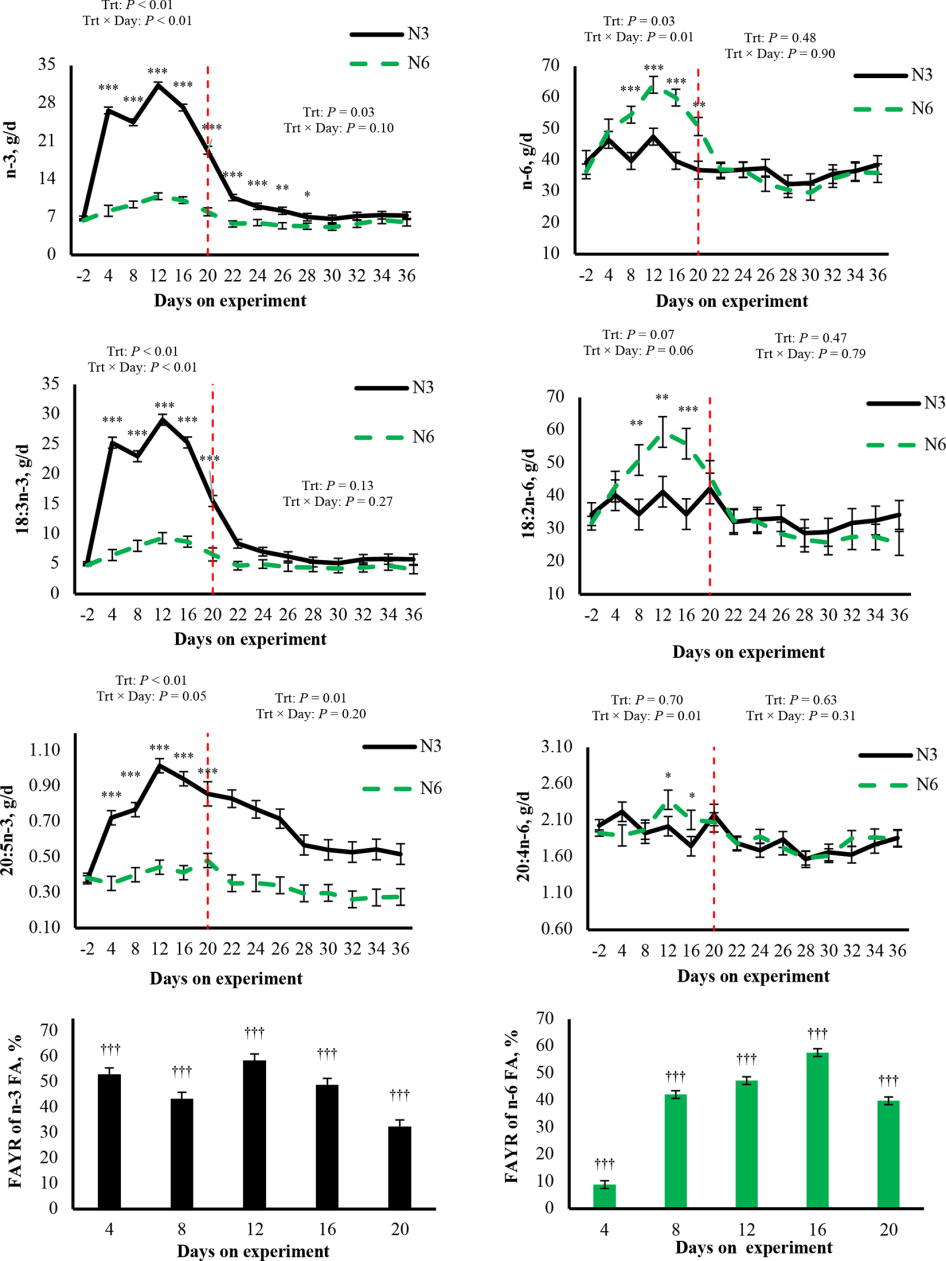
Yields (g/d) of total n-3 and n-6 FA, 18:2n-6, 18:3n-3, 20:4n-6, and 20:5n-3 in milk fat of cows during treatment (from d 1 to 20) and carryover periods (from d 21 to 36; treatment and carryover periods separated by red dashed line). Treatments were abomasal infusions of ∼43 g/d of 18:2n-6 (N6) or 18:3n-3 (N3). Trt × Day = interaction between treatments and day. When the interaction tended to be significant (*P* ≤ 0.15), comparisons were performed between treatments within each day with tendencies at *P ≤ 0.10; and significances at **P ≤ 0.05 and ***P ≤ 0.05. Error bars represent SEM. Bar plots represent milk FA yield response to additional FA (FAYR, %) of cows during treatment (from d 1 to 20) and carryover periods (from d 21 to 36). Intercepts for each day were compared against zero with significance at †††*P* ≤ 0.01. Error bars represent SEM.

We observed interactions or tendencies for interactions between treatment and time for the contents of total n-3 FA, total n-6 FA, 18:2n-6, 18:3n-3, 20:4n-6, and 20:5n-3 (*P* ≤ 0.12, Figure 2). Compared with N6, N3 increased the contents of total n-3 FA (1.09 g/100 g), 18:3n-3 (0.98 g/100 g), and 20:5n-3 (0.02 g/100 g) from d 4 to 20 (*P* < 0.01). Compared with N3, N6 increased total n-6 FA from d 4 to 20 (1.60 g/100 g), 18:2n-6 from d 4 to 16 (1.61 g/100 g), and 20:4n-6 from d 12 to 16 (0.04 g/100 g, *P* ≤ 0.02, Figure 2). We observed a main effect of treatment without interaction, with N3 increasing milk fat content of 22:5n-3 (0.01 g/100 g, *P* = 0.01) compared with N6 (data not shown).

**Figure 2:**
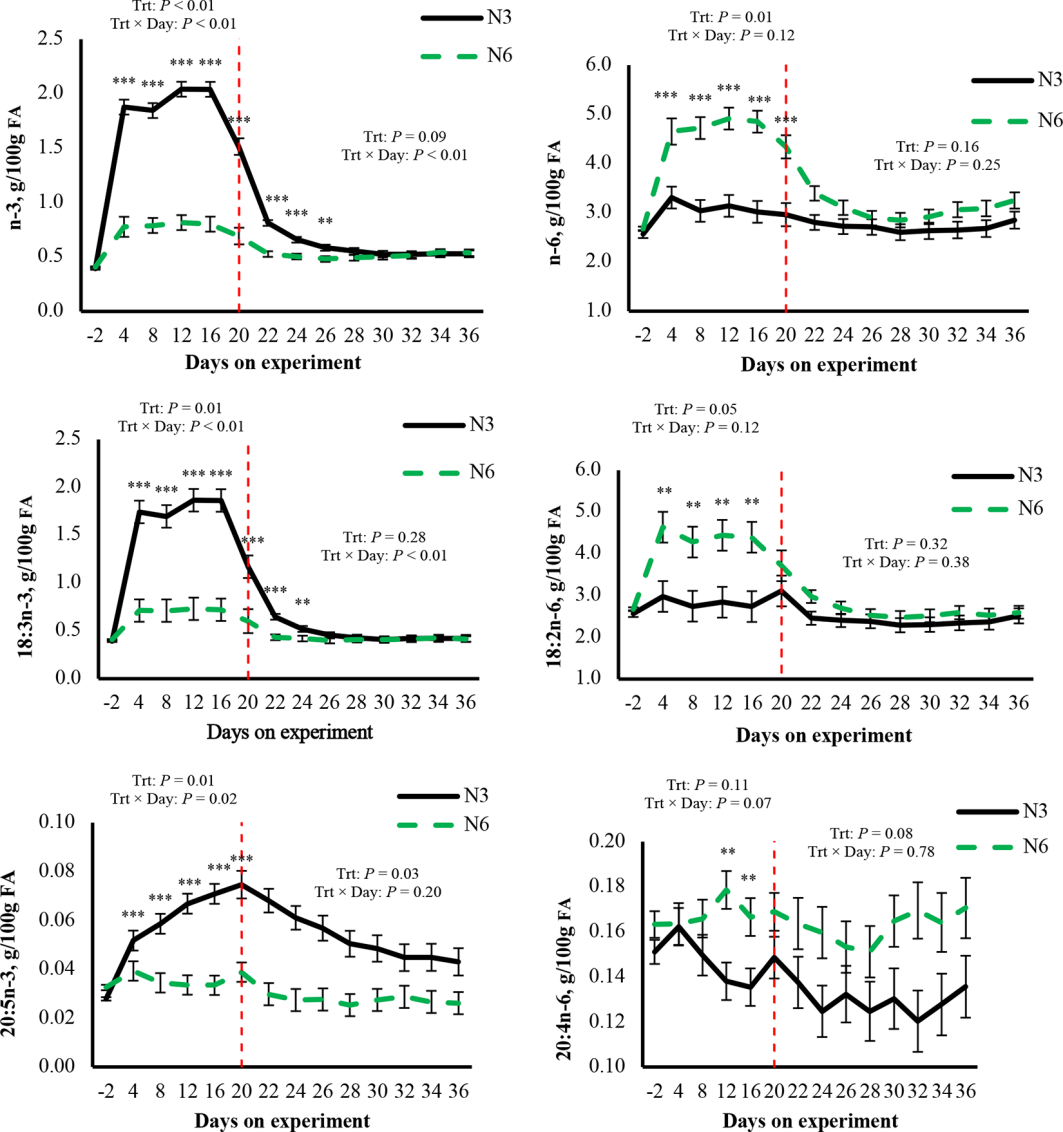
Contents (g/100 g FA) of total n-3 and n-6 FA, 18:2n-6, 18:3n-3, 20:4n-6, and 20:5n-3 in milk fat of cows during treatment (from d 1 to 20) and carryover periods (from d 21 to 36; treatment and carryover periods separated by red dashed line). Treatments were abomasal infusions of approximately 43 g/d of 18:2n-6 (N6) or 18:3n-3 (N3). Trt × Day = interaction between treatments and day. When the interaction tended to be significant (*P* ≤ 0.15), comparisons were performed between treatments within each day with significances at **P ≤ 0.05 and ***P ≤ 0.01. Error bars represent SEM.

During the carryover period, we did not observe any main effect of treatment on production responses (*P* ≥ 0.31), with no interactions between treatment and time (*P* ≥ 0.17, data not shown). Overall, we observed the following means (± SD): 28.6 ± 3.34, 36.8 ± 5.74, 1.34 ± 0.55, and 1.16 ± 0.27 kg/d, and 3.65 ± 0.55, and 3.22 ± 0.66 g/100 g for DMI; the yields of milk, milk fat, and milk protein; and the contents of milk fat and milk protein, respectively.

We observed an interaction between treatment and time (*P* = 0.10, Figure 1), with N3 increasing or tending to increase the yield of total n-3 FA from d 22 to 28 (3.08 g/d, *P* ≤ 0.08, Figure 1). We observed a main effect of treatment without interaction, with N3 increasing the yield of 20:5n-3 (0.32 g/100 g) compared with N6 (*P* = 0.01, Figure 1).

We observed interactions between treatment and time for the contents of total n-3 FA and 18:3n-3 (*P* < 0.01, Figure 2). Compared with N6, N3 increased total n-3 FA (0.17 g/100 g) from d 22 to 26 and 18:3n-3 FA (0.16 g/100 g) from d 22 to 24 (*P* ≤ 0.03, Figure 2). We observed some main effects of treatment without interactions. Compared with N6, N3 increased or tended to increase the contents of 20:5n-3 (0.02 g/100 g, *P* = 0.03, Figure 2) and 22:5n-3 (*P* = 0.07, data not shown). Compared with N3, N6 tended to increase the content of 20:4n-6 (*P* = 0.08, Figure 2). It is important to note that we did not detect 22:6n-3 in milk fat during either the treatment or carryover periods.

Our results show that abomasal infusions of N3 and N6 increased the yields and contents of n-3 and n-6 in milk fat during the treatment period, respectively. Interestingly, N3 increased the yields and contents of 20:5n-3 and 22:5n-3 in milk, but we did not detect 22:6n-3, highlighting that the conversion of 18:3n-3 to 22:6n-3 is very inefficient, as discussed in our companion paper (dos Santos Neto et al., 2024). This also agrees with a review by Moallem (2018), in which the author did not detect 22:6n-3 in milk fat in any of the experiments supplementing dairy cows with flaxseed products. Interestingly, a recent study by Gervais et al. (2023) that infused high doses of flaxseed oil (0, ∼70, ∼140, ∼280, and ∼560 g/d) detected 22:6n-3 in milk fat but found no effect of treatment on this FA.

Consistent with our observations in plasma lipid fractions (dos Santos Neto et al., 2024), the relative increase in n-3 milk FA was higher than for n-6 milk FA. Considering yield differences, N3 increased total n-3 FA by 16.5 g/d and N6 increased n-6 FA by 13.7 g/d. Furthermore, carryover effects were observed for n-3 but not n-6 milk FA. This is consistent with our companion study, where we observed a positive carryover effect for n-3 but not n-6 FA in plasma TG and NEFA (dos Santos Neto et al., 2024). Although these lipid fractions were not the most responsive to our treatments, they are the fractions that predominantly deliver FA to the mammary gland (dos Santos Neto et al., 2024; Christie, 1981). In addition, these results could be associated with the preference of Δ^6^-desaturase for 18:3n-3 over 18:2n-6 during the first step in the biosynthesis pathway of longer n-3 and n-6 FA (Palmquist, 2009). We observed higher FAYR for n-3 FA compared with n-6 FA during the treatment period. Notably, FAYR for n-6 FA was 39%, lower than the 46% reported by Drackley et al. (1992) when abomasally infusing over 5 times more 18:2n-6 than our current study (240 vs. 43 g/d). Urrutia et al. (2023) abomasally infused single bolus doses of ∼49 or 80 g/d of 18:3n-3 and observed FAYR of 59 and 43%, respectively. We observed an FAYR average of 47% for n-3 FA when infusing 43 g/d of 18:3n-3,

The actual yields of n-3 FA (N3 = 25.8 g/d and N6 = 9.28 g/d) were lower than those of n-6 FA (N3 = 42.1 g/d and N6 = 55.8 g/d). Our diets had slightly higher levels of 18:2n-6 (1.09% diet DM) than 18:3n-3 (0.90% diet DM, dos Santos Neto et al., 2024). As reported previously, Δ^6^-desaturase has a greater affinity for 18:3n-3 than 18:2n-6. However, 18:2n-6 is normally present at higher concentrations in cellular pools than 18:3n-3, leading to a greater synthesis of long-chain n-6 FA (Burdge and Calder 2006; Palmquist, 2009), which reflects the higher yield and content of total n-6 compared with total n-3 milk FA in the present study. A recent study by Sun et al. (2023) observed similar responses in milk FA profile of transition cows fed an n-6 FA-enriched diet based on extruded soybean compared with an n-3 FA-enriched diet based on extruded flaxseed.

It is important to emphasize that we abomasally infused the treatments, avoiding ruminal biohydrogenation and ensuring the PUFA were delivered to the small intestine. In practical terms, increasing n-3 FA in milk by feeding 18:3n-3 to dairy cows on farms is challenging. A meta-analysis evaluating feeding studies that included 6 different dietary sources of flaxseed reported a transfer efficiency varying from ∼2% to 6% (Leduc et al., 2017). In this study, the authors did not estimate FAYR, but simply calculated the grams of FA in milk per 100 g of FA consumed. If we use the same approach and consider the basal diet supply of n-3 FA along with the N3 infusion, only ∼9% of the total n-3 FA provided during the treatment period were incorporated into milk fat. An additional difficulty for enriching n-3 FA in milk is associated with their storage and transport dynamics, where absorbed PUFA are not readily incorporated into lipid fractions that effectively deliver FA to the mammary glands (Christie, 1981). As we reported in our companion paper, plasma TG and NEFA were the least responsive lipid fractions to abomasal infusions of N3 or N6. Plasma PL and CE had the highest PUFA content and were the most responsive fractions to these abomasal infusions (dos Santos Neto et al., 2024). Selectively incorporating essential FA into PL and CE ensures their use for vital biological mechanisms and reduces their use in nonessential roles, such as milk fat (Mattos and Palmquist, 1977; Christie et al., 1986). Gervais et al. (2023) reported it is possible to overcome this mechanism in cows and increase 18:3n-3 in TG at the expanse of CE when abomasally infusing very high levels of flaxseed (∼140 to 558 g/d). However, at ∼558 g/d, milk was prone to oxidative degradation (Rico et al., 2023). Therefore, strategies to increase the absorption of PUFA focusing on beneficiating the cow itself rather than on the enrichment of specific FA for human consumption should be more successful.

Numerous studies have reported that reducing the dietary n-6 to n-3 FA ratio attenuates inflammation and improves production and reproduction of dairy cows (Greco et al., 2015; Sinedino et al., 2017; Sun et al., 2023). Our current study was primarily designed to examine alterations in blood plasma lipids and milk FA profile; it was not designed to evaluate production variables, which did not differ between treatments. In addition, this could be related to the fact that we used late-lactation cows (252 ± 33 DIM).

In conclusion, abomasally infusing N3 and N6 for 20 d increased the yields and contents of n-3 and n-6 FA in milk, respectively. The relative increases were more pronounced for n-3 than for n-6 milk FA. During the treatment period, N3 had a n-3 FA transfer efficiency to milk of 47%, whereas the transfer efficiency of n-6 FA for N6 was 39%. We did not detect 22:6n-3 despite N3 increasing 20:5n-3 and 22:5n-3. Furthermore, a positive carryover effect was only observed for n-3 FA.
